# Potential role of glucagon like peptide 1 in taste receptors

**DOI:** 10.3389/fendo.2025.1683419

**Published:** 2026-01-23

**Authors:** Jose Luis Eduardo Doval-Caballero, Aldo Ferreira-Hermosillo, Genesis Dinora Eugenio-Ponce, Manuel Ramon García-Sáenz, Raúl Ibarra-Salce, Andrea Patricia Tenorio-Rojo, Eduardo Salif Luna-Avila, Paulo César Gete-Palacios, Fernando Pérez-Hernández, Eduardo Rojas-Milán, Luis Angel López-Cruz, César Alejandro Méndez-Hernández

**Affiliations:** 1 Obesity Clinic, National Institute of Medical Sciences and Nutrition Salvador Zubirán, Mexico City, Mexico; 2 Unidad de Investigación Médica en Enfermedades Endocrinas, Instituto Mexicano del Seguro Social, Mexico City, Mexico; 3 Servicio de Endocrinología, Instituto Mexicano del Seguro Social, Mexico City, Mexico; 4 Universidad Autonoma de Coahuila, Saltillo, Mexico

**Keywords:** food preferences, gastrointestinal hormones, glucagon-like peptide 1, gut-brain axis, obesity, taste, taste receptors

## Abstract

The perception of taste is a complex physiological process that extends far beyond the simple detection of flavor molecules, serving as a critical interface between nutrient sensing, metabolic regulation, and feeding behavior. Emerging evidence reveals that this process is profoundly modulated by endocrine and neuromodulatory systems, creating a sophisticated gut-brain-taste axis that integrates peripheral gustatory signals with central homeostatic and hedonic mechanisms. Hormones such as glucagon-like peptide-1, leptin, ghrelin, and CCK not only regulate appetite and energy balance but also directly influence taste receptor expression and function in the tongue and gastrointestinal tract. Concurrently, neuromodulators like dopamine, serotonin, and norepinephrine fine-tune taste sensitivity at both peripheral (taste buds) and central (reward circuitry) levels, linking chemosensation to motivational states. These interactions are further complicated by metabolic conditions such as obesity and diabetes, where hormonal resistance (e.g., leptin, insulin) and neurotransmitter dysregulation contribute to altered taste preferences and compulsive eating behaviors.

## 1 Introduction

Taste perception represents a sophisticated biological system that transcends mere flavor detection, functioning as a vital nexus connecting nutritional sensing, metabolic control, and eating patterns. Current research demonstrates that this sensory modality is significantly influenced by both hormonal and neural regulatory networks, establishing an intricate gut-brain-taste axis that synchronizes peripheral taste signals with central mechanisms governing physiological balance and food reward. Key metabolic hormones (including glucagon-like peptide-1 (GLP-1), leptin, ghrelin, and cholecystokinin (CCK) play dual roles: not only regulating hunger and metabolism, but also directly modulating the activity and expression of taste receptors throughout the oral cavity and gastrointestinal tract. At the same time, neurotransmitters such as dopamine, serotonin, and norepinephrine finely tune taste responsiveness from individual taste buds to cortical reward pathways, ensuring adaptive eating behaviors (1, 2). In certain metabolic disorders, such as obesity and type 2 diabetes, exists a disruption of these finely coordinated systems, where impaired hormonal sensitivity (particularly to leptin and insulin) and altered neurotransmitter function lead to distorted taste perception and maladaptive food preferences (3).

The process of food intake, digestion and nutrient absorption is tightly coordinated through bidirectional signaling between the gastrointestinal tract and the central nervous system (CNS), collectively referred to as the gut-brain axis (GBA). This constant dialogue involves a network of neural, endocrine, immune, and metabolic signals that inform the brain about the body’s nutritional status, influencing appetite, satiety, and reward pathways (3–5). Hormones secreted in response to nutrient ingestion, such as incretins GLP-1 and glucose-dependent insulinotrophic polypeptide (GIP), mediate satiety, regulate glucose metabolism, and modulate central reward pathways, contributing to the regulation of both energy homeostasis and food preference (6–8).

Historically, taste perception was regarded as a mechanism for flavor categorization. However, modern evidence reveals that the gustatory system is a dynamic neuroendocrine system, crucial for nutritional sensing, metabolic regulation, and behavioral adaptation (1, 2, 9) The tongue and gastrointestinal tract jointly form the first step of nutrient sensing within the broader GBA, translating chemical stimuli into hormonal and neural signals that shape hunger, satiety, and hedonic reward (1, 3). As the understanding of obesity has evolved, now being recognized as a multifactorial disease involving both homeostatic and hedonic control of hunger (10, 11); the gustatory system has gained new relevance. This is especially relevant in obesogenic environments such as modern diets, rich in ultra-processed and high-caloric foods, which have contributed to nearly 45% of the global adult population living with overweight or obesity (12–14).

Built on this paradigm, a growing body of evidence indicates that GLP-1 acts beyond its classical endocrine role, directly influencing the gustatory system. Previous reviews (15) first proposed that GLP-1 and GLP-1 receptor agonists (GLP-1RAs) may modulate taste perception, especially for sweet stimuli. Our review expands upon this foundational work by incorporating post-2020 findings, emphasizing the bidirectional communication between taste bud cells (TBC) and gut endocrine signaling, and critically analyzing the potential clinical implications of GLP-1RAs on taste perception, food reward, and adherence to obesity treatment.

Therefore, the purpose of this review is to synthesize and contextualize the evidence that links taste perception and gut-derived peptides, with a particular focus on the emerging role of GLP-1 as both a metabolic hormone and a neurosensory modulator. We aim to elucidate how GLP-1 participates in the early stages of taste perception, the molecular and neural mechanisms underlying this process, and its broader implications for understanding the pathophysiology of obesity and the therapeutic effects of GLP-1RAs. For this purpose, we searched Google Scholar, PubMed, EBSCOhost and WEB of Science electronic databases for publications up to September 2025. The MeSH terms included: (Glucagon like peptide 1), (taste, receptors), (gastrointestinal hormones), (obesity), (food preference), (gut-brain axis), (sweet taste), (umami), (bitter taste) and (taste bud cells). Publications including preclinical and clinical evidence of GLP1, GLP1RAs and taste perception published in English and Spanish language were included. We used thematic content analysis to extract information, and presented a narrative review account of the findings.

## 2 The taste sense: physiology and integration

### 2.1 The tongue: structure and innervation

The Taste Bud Cells (TBC) are sensory organs with a bulb-shaped structure that possess taste receptor cells (TRC). As far as we know, and understanding the wide interindividual variation, there are approximately 2000 to 5000 TBC distributed on the soft palate, pharynx, larynx and upper esophagus, but mainly localized in the tongue (16, 17).

**Figure 1:**
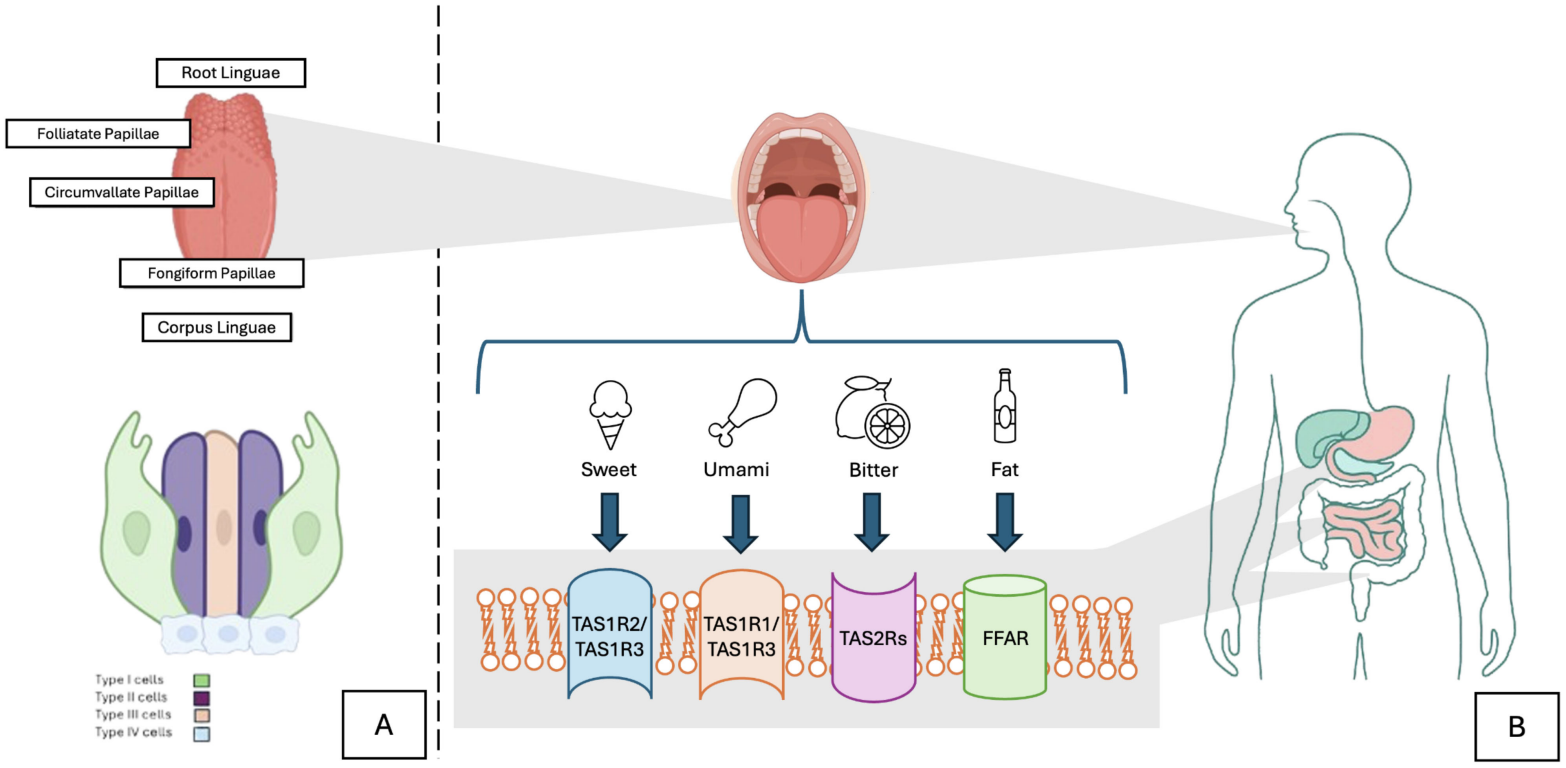
**(A)** Scheme of the tongue and the distribution of taste bud cells (TBC) across Papillae. TBC are sensory organs with a bulb-shaped structure in which different type of specialized cells resides (TRC). **(B)** TRC are structured as mentioned in this figure: Type I cells function as supporting cells; while Type II cells (key operator) have G-protein receptors that detect different kinds of flavors depending on the receptor it is attached (TAS1 R1,R2,R3 and TAS2R) and Type III cells function as presynaptic cells that form conventional neuronal synapses with sensory afferent nerve fibers, releasing serotonin, norepinephrine, and GABA as neurotransmitters. These same receptors in the gut lead to hormone secretion through the enteroendocrine cells, having an important regulation on appetite and metabolism (15, 21, 24, 25).

The tongue consists of two parts: the anterior two-thirds referred to as the *corpus linguae* (body) and the posterior third known as the *radix linguae* (root), both of which are separated by a V-shaped groove called *terminal sulcus*. The root of the tongue connects it to the jaw and to the hyoid bone, while the body stores on its surface four distinct lingual papillae, each with a unique anatomical and functional characteristic and an individual relationship with taste perception (18, 19): The filiform papillae, although the most numerous, have no gustatory functionality, but provide texture sensation and assist in the mechanical manipulation of food. The circumvallate papillae, arranged in a “V” shape at the base of the tongue and innervated by the glossopharyngeal nerve (IX), contains the highest concentration of taste buds in the tongue. The fungiform papillae, located at the tip and edges of the tongue, contain taste buds richly vascularized and are innervated by the chorda tympani, a branch of the facial nerve (VII), and the foliate papillae, located on the posterior lateral folds, which apparently have less population of TBC with age (20–23). (**Figure 1**) It is also important to highlight the complex neuronal architecture of the tongue, as we should consider three different innervation types: 1) Autonomic innervation: generates the salivatory stimuli and regulation of local blood flow, that plays an important role in food pleasure and avoidance (26) 2) Motor innervation: control of muscle movement, such as mastication, swallow, speech, and breathing (27), and 3) Somatosensorial innervation: that encompasses taste perception, nociception and proprioception (18). As stated above, it is innervated by branches of three cranial nerves (CN); the chorda tympani and greater superficial petrosal branches from the facial nerve (CN VII); the lingual branch provided by glossopharyngeal nerve (CN IX), and the superior laryngeal branch part of the vagal nerve (CN X) (18, 21). All the information, related to attributes of tastant quality, intensity, and hedonic quality travels through the afferent nerve fibers from the TBC and guided by these CNs converge in the gustatory portion of the nucleus of the tractus solitarius (NTS), that subsequently, will move through the thalamus, limbic system, and insula in the gustatory cortex (18, 21, 28). In humans, the NTS establishes direct to the ventral and posteromedial (VPM) nucleus of the thalamus (29). In addition to the mentioned cranial nerves, somatosensory innervation is also performed by the trigeminal nerve (CN V), which consists of three main branches, ophthalmic, maxillary, and mandibular. Here, the information its first stored in the trigeminal ganglia, subsequentially ascending through axonal projections that enter to the pontine nucleus (or main sensory nucleus) and project to the VPM, converging in the sensory cortex in the postcentral gyrus, where information about touch-position and pain-temperature sensation are integrated; as an example, the lingual branch of the mandibular nerve transmits sensorial information such as pain, thermal sensitivity, and mechanical stimuli through the *C, A-δ, and A-β* fibers (28, 30). In addition to this, the trigeminal perception of the peripheral and somatosensory systems can be guided through transient receptor potential channels (TRP), that are cation selective (mainly calcium) and modulated by calmodulin, phospho-lipases, and kinases (31). These channels affect gustatory processing by altering sensibility to changes in temperature (activated indirectly by tastants or directly by certain spices) in response to physical stimuli, such as mechanical forces and temperature, and by chemical agents (chemestesis), evoking sensations of touch, temperature, and pain, depending on the kind of subunit they are attached to; for example, TRPs help humans perceive two kinds of tastes at the same time, like sweet taste, but at the same time, the lasting sensation of coolness that menthol has (perception named as pungency) (32–34). In this complex interplay, we can assume that some neurons respond to particular combinations of taste and texture produced by food, helping us to decipher pleasant stimuli, reward, and appetite control (35, 36). Therefore, we can state that the sensation and perception of food texture (primarily associated with filiform papillae), may outperform as well or better than the fingertips; and as a matter of fact, can be extremely important for the preference and attitude toward different kinds of food (37, 38).

### 2.2 Taste receptor cells

**Figure 2:**
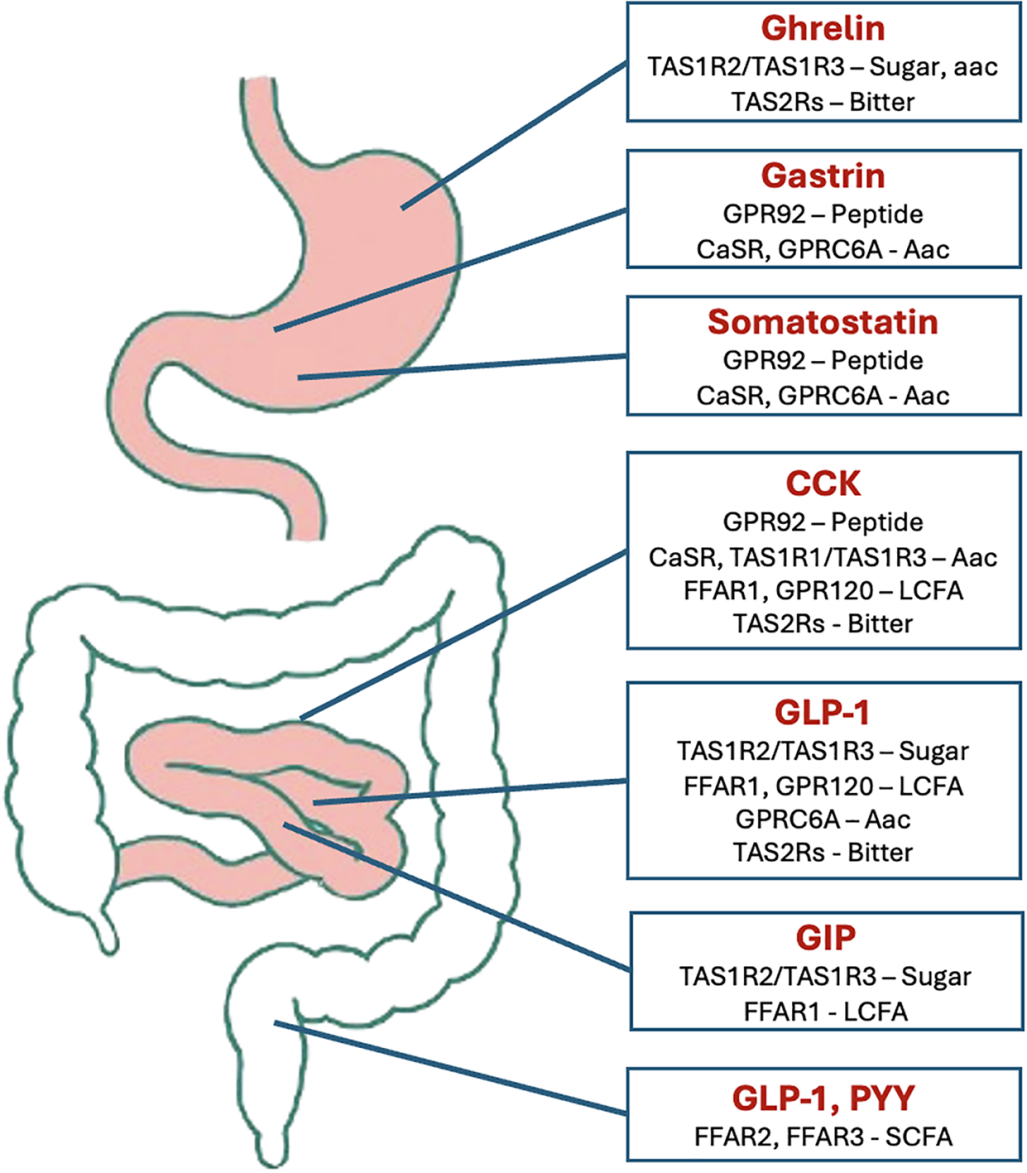
Taste receptors and enteroendocrine cells location and relationship. AAC, Aminoacids; CaSR, Calcium-sensing Receptor; FFAR, Free fatty Acid Receptor; GPR, G-protein receptor; TASR, Taste Receptor.

Taste buds are composed of distinct types of cells, some of them functioning as support cells, while there are others more specialized, named Taste Receptor Cells (TRC), which in turn, are divided in four categories. Each TBC category has approximately 50–100 different kinds of TRCs (24, 25). This classification arises from their earliest histological identification through electron microscopical studies; however, physiologically we can classify two subtypes of TRCs. Cells that respond to taste stimulation, such as sensory receptors cells with subsequent transduction cascades (such as Type II); and cells excited by depolarization, but not by taste stimulation (such as Type I and III) (39) (**Figure 2**).

#### 2.2.1 Type I cells

Also known as glial cells, they comprise 50% of the total population of TBC. They have a resembling role as “supporting cells”, as they wrap around type II and III cells, forming lamellar processes that may facilitate the degradation of neurotransmitters, and also creating the independent clusters of each TBC (28, 40, 41). This process may be generated by limiting ATP diffusion, stopping taste stimulation spread by cell expression of ecto-ATPase, providing a more protected environment for cell-cell communication (42). These cells also regulate the ionic environment, altering salt-taste perception through positive amiloride-sensitive response to NaCl by the epithelial sodium channels (ENaC), which are sensitive to low sodium concentrations (43, 44).

#### 2.2.2 Type II cells

Although they are a relatively small subpopulation (only about 19-25% of all TRCs), they might be considered as the key receptor cells. Type II cells express G protein-coupled receptors (GPCRs) from the type C family, along with metabotropic glutamate and gamma-aminobutyric acid B receptors (45, 46). These receptors function as monomers or dimers. The receptor type 1 (TAS1R) aggregates as heteromers, with different combinations that recognize specific classes of tastes: TAS1R2 + TAS1R3 for sweet and TAS1R1 + TAS1R3 for umami. The TAS2R receptor (with more than 25 variants characterized in humans) recognizes bitter stimuli. These receptors activate intracellular cascades mediated by gustducin, phospholipase C beta 2 (PLCβ2), inositol triphosphate (IP3), and Transient Receptor Potential Melastatin 5 (TRPM5), leading to the release of ATP as a neurotransmitter (2, 24, 47). This molecular machinery mirrors the one found in enteroendocrine cells of the gut, indicating that similar receptors operate in different physiological contexts. However, their signaling diverges at later stages: in the tongue, activation of these receptors produces neurotransmission and conscious taste perception, whereas in the intestine, the same receptor types modulate hormone secretion (such as GLP-1 and GIP) through endocrine signaling pathways (48, 49).

#### 2.2.3 Type III cells

They have structural features shared with the other two types of cells, representing 15% of total TRCs (50). These cells function as presynaptic cells that form conventional neuronal synapses with sensory afferent nerve fibers, releasing serotonin, norepinephrine, and GABA as neurotransmitters (2, 51, 52). They are also representative for the detection of sour taste and are responsible for signals that prevent the ingestion of unripe, spoiled, or fermented food. This is allowed by the Otopetrin-1 (OTOP1) channel and the expression of the polycistin-2-like 1 (Pkd2l1) gene, which allows proton entry, and generating lower intracellular pH (around 3.9) to block K+ channels and depolarizing the TRCs (53–55).

#### 2.2.4 Type IV cells

They were originally referred as basal cells because of their location in the tongue; its existence has been controversial and it is thought that these cells act as progenitors, constantly renewing the gustatory epithelium during the synthesis phase of TBC renewal, nonetheless, there are many questions regarding its cycling, especially in rodents (24, 56).

The shared receptor families (T1Rs and T2Rs) illustrate a common evolutionary template for chemosensory signaling between the oral cavity and the gastrointestinal tract. The oral pathway mediates rapid neural transmission, while the intestinal pathway mediates slower hormonal responses, both converging on the GBA (57, 58).

### 2.3 Role of taste receptors in the gastrointestinal hormone secretion

Although, we commonly associate TRCs and TASR only to the oral cavity, they are also localized in extra-oral tissues, including the gastrointestinal tract, where they participate in energy homeostasis by modulating enteroendocrine hormone release (GLP-1, GIP, CCK, PYY) and nutrient sensing, thereby influencing gastric emptying and metabolic adjustments (e.g., glycemia) (15, 59, 60). For clarity, we summarize connections between taste-like receptors and gut hormone production (**Table 1**, **Figure 2**).

**Table 1:** Taste receptors and relation with gut hormone secretion and nutrient stimulus.

Taste receptor (GPCR)	Nutrient stimulus	Hormones	Enteroendocrine cells
TSR1R2/TSR1R3	Glucose, sweeteners	GLP-1, GIP, PYY	L cells, K cells
TSR1R1/TSR1R3	Amino acids	CCK, GLP-1	I cells, L cells
TSR2Rs	Bitter compounds	CCK, Ghrelin, GLP-1, PYY	Various enteroendocrine cells
GPR120	Unsatured fatty acids	GLP-1, CCK	I cells, L cells
FFAR1	Long-chain fatty acids	GLP-1, GIP	K cells, L cells
FFAR2, FFAR3	Short-chain fatty acids	GLP-1, PYY	L cells

#### 2.3.1 Sweet and umami receptors (TAS1Rs)

L and K cells in the intestine express taste receptors that sense luminal carbohydrates and amino acids, triggering secretion of gut hormones such as GLP-1 and GIP. Studies in mice show that sucrose and umami stimuli can promote GLP-1 secretion from taste bud-like cells, reinforcing the link between nutrient detection and enteroendocrine signaling (61). At the same time, sucrose, artificial sweeteners, and umami stimuli promote NPY and GLP-1 secretion from mouse circumvallate taste buds (62). Studies in both humans and mice have shown that long-chain free fatty acids (LCFAs) increase GLP-1 secretion from taste cells, further reinforcing sucrose preference in a GLP-1 receptor-dependent manner (63, 64). These findings reflect the dependence on GLP-1 for downstream signaling, cell depolarization, and transmission of signals to GLP-1Rs in sensory input for the full perception of taste. Glucagon and its receptor are present in a subpopulation of type II cells; and evidence exists that pharmacological or genetic disruption of glucagon signaling in gustatory cells leads to a reduction in sweet taste signaling (65). Paracrine glucagon signaling through its specific receptor enhances sweet signaling in a local circuit (29, 66). In contrast to the effects of sweet taste on GLP-1 release, glucagon secretion decreases in response to sweet taste perception, indicating that glucagon is expressed in a different population of type II taste buds than those that express GLP-1 and GLP2 (67).

Umami taste receptors (TAS1R1/TAS1R3 heterodimers) also respond to amino acids such as glutamate and aspartate. In mice, activation of this receptor has been associated with CCK secretion. On the other hand, additional studies in ghrelin-producing cell lines have demonstrated ghrelin release in response to monosodium glutamate and L-alanine through these receptors. There are some studies using lactisole, a sweet taste receptor antagonist, that have shown reduced GLP-1 and PYY secretion in response to glucose, but no effect on CCK release (68–70).

#### 2.3.2 Bitter receptors (TAS2Rs)

Bitter taste receptors are involved in detecting potentially harmful substances. TAS2R5 has been identified in L cells that secrete GLP-1, TAS2R38 is expressed in enteroendocrine cells (EECs) and has been related to CCK, PYY, and GLP-1 secretion (71, 72). CCK functions in an autocrine manner by binding to the cholecystokinin type A receptor (CCK-AR), inhibiting channel activation and increasing intracellular Ca2+ by up to 30% upon TAS2R stimulation (73). Because K+ channels are required for membrane repolarization in TASR, these cells remain in a depolarized state for an extended period, resulting in prolonged bitter signaling (2, 29). In animal experiments, rats lacking CCK type 1 receptors have shown to improve sensitivity to sweet substances, including artificial sweeteners, and the absence of this receptor alters sweet taste perception (74). In mice, intragastric administration of bitter compounds increased ghrelin levels and food intake. *In vitro* studies have shown that monomeric flavanols stimulate ghrelin secretion, while oligomeric flavanols and gallic acid inhibit it (75–77).

#### 2.3.3 Extra-endocrine expression of taste receptors

Beyond EECs, it is also important to mention that taste receptors may also be present in the brush cells, a subgroup of solitary chemosensory cells found in the nasal cavity and other regions of the gut, especially where food passes from the fundus to the corpus, and digestive process are initiated, where expression of TAS1R1, TAS1R3, and a-gustducin are involved, suggesting a role in amino acid sensing (60, 78, 79). In the colon, TAS1Rs have been localized in Paneth cell secretory granules, coexpressing with a-transducin, indicating a chemosensory role (60). In mice, TAS2R131 has been implicated in xenobiotic defense by promoting mucus production in the colon (80).

## 3 Gastrointestinal hormone secretion

The GBA is a bidirectional communication system formed by the CNS and the gastrointestinal tract, involving humoral pathways (gut-brain peptides, gut microbial metabolites, and cytokines) and neural pathways mediated by the vagus, spinal nerve, and the autonomic nervous system; their interaction adjusts physiological functions such as behavioral regulation and immune response, and as mentioned before, may have a close interaction with taste cells and their receptors (15, 29, 81, 82). The process of food intake, previously seen as a purely physiologic state of energy homeostasis, is a product of a complex interaction that starts with stomach mechanoreceptors, involving peptide release for stimuli of hunger and satiation (depending on food quantity), peristalsis and gastric accommodation (83–87). The EECs are the main cells involved in the production and secretion of a variety of hormones and signaling molecules in the GBA; and even as they account for less than 1% of the intestinal epithelium, they establish a complex signaling network, that is likely to be the first step of integration, involving the information obtained from the gut lumen (63, 88, 89). Traditionally, the EECs were classified by the peptide they secrete, although more recently, application of RNA sequencing has led us to know that a single EEC can produce multiple hormones within an individual, and different hormonal promoters may be active at different times (90, 91) The distribution of the different EECs across the gastrointestinal tract that is generally accepted is: Stomach: P/D1 cells (ghrelin), D cells (somatostatin), G cells (gastrin), and ECL cells (histamine). Small intestine and colon: I cells (CCK), K cells (GIP), and L cells (GLP-1 and PYY). All these cells express chemoreceptors that detect luminal contents and initiate hormone release, influencing food intake, insulin secretion, and intestinal motility (92–94).

### 3.1 Alternative nutrient-sensing mechanisms

Some nutrients stimulate intestinal hormone secretion independent of taste receptors, via pathways involving other GPCR or nutrient transporters (95, 96):

- Stomach: Calcium-sensing receptor (CaSR) and GPRC6A detect a broad range of amino acids and calcium, promoting gastrin and somatostatin release (89, 97).
- Duodenum: Free fatty acid receptor (FFAR) 1 and CaSR, expressed on I cells, mediate CCK secretion in response to fatty acids and amino acids (98, 99).
- Jejunum and ileum: Sodium-glucose cotransporter 1 (SGLT1) and ATP-sensitive potassium channels (KATP) play critical roles in GLP-1 secretion. KATP inhibition enhances GLP-1 release, while SGLT1 is involved in glucose-triggered secretion (100).
- K and L cells: Express FFAR1, which is activated by long-chain fatty acids, promoting GIP and GLP-1 secretion (99).
- Colon: FFAR2 and FFAR3 detect short-chain fatty acids, enhancing PYY secretion (101).

## 4 Hormonal taste alterations and central nervous system signaling in obesity

### 4.1 Hormonal-induced taste alterations in obesity

Certain hormones are involved in modulating the sense of taste. The binding of leptin to its receptor (Ob-R) in type II TRCs, activates K_ATP_ channels, hyperpolarizing the cell in response to sweet stimuli. In leptin-sensitive individuals, this signaling may help prevent the excessive intake of sweet foods. However, in states of leptin resistance such as obesity, this regulatory mechanism may be impaired (102, 103). On the other hand, adiposity is associated with a state of low-grade chronic inflammation, which can interfere with the survival and renewal of taste buds (104, 105). In 2009, Wang et al. identified the expression of Toll-like receptors (TLRs) and interferon receptors (IFN) in TRCs, which, upon activation, trigger apoptosis and disrupt cellular balance in taste buds. Since then, it has been hypothesized that acute inflammation can reduce the population of taste cells, affecting taste discrimination (106). In 2018, Kaufmann et al. analyzed the inflammatory response triggered by a high-fat diet in mice. They found increased expression of TNF-α in the taste epithelium, as well as altered expression of TNF-α receptors 1 and 2. These changes were associated with a lower density of taste buds in the circumvallate region. However, the balance between type I, II, and III cells was not affected. Additionally, fewer Ki67-immunoreactive cells were observed, suggesting a lower density of taste progenitor cells (105). Recently, Archer et al. studied transcriptomic profile differences in taste buds between lean and obese individuals. Their analysis revealed a decrease in the expression of SHH (Sonic hedgehog gene) and PLCβ2 (Phospholipase C Beta 2) in obese subjects. SHH has previously been identified as a critical signal for the maintenance and renewal of taste cells, while PLC-β2 is a marker involved in sweet, umami, and bitter signaling within type II cells, which may contribute to reduced sensitivity to taste stimuli (107). This information supports the hypothesis that dysfunction in obese individuals may originate from changes in taste bud morphology, which result from a reduction in taste bud abundance and altered signaling (104). Finally, several studies on post-bariatric surgery patients (Roux-en-Y gastric bypass) have observed a decreased preference for highly concentrated sweet foods, without significant changes in response to salty, sour, or bitter stimuli. This phenomenon may be explained by alterations in hormonal secretion induced by bariatric surgery (108, 109).

### 4.2 Central nervous system adaptations to taste in obesity

Certain brain regions are responsible for processing sensory information through visual, olfactory, and auditory pathways, as well as gustatory signals. The integration of gustatory sensory signals occurs primarily in the orbitofrontal, prefrontal, and insular cortex (110). Meanwhile, “food memories” are distributed across a neural network involving the hippocampus, prefrontal cortex, dorsal striatum, and amygdala structures (111–113). Once food passes through the gastrointestinal tract (GI), taste receptors, along with mechanosensors and chemoreceptors within the GI tract, transmit information via afferent branches of the vagus nerve and hormonal signaling (e.g., GLP-1, GIP, PYY, insulin, among others) (46). Multiple studies have established that the hypothalamus serves as the central integrative hub for satiety and appetite regulation. This is exemplified by experimental models showing that selective ablation or stimulation of Agouti-related peptide (AgRP)/NPY/GABA neurons in the basomedial hypothalamus leads to either starvation or hyperphagia with subsequent obesity, respectively (114, 115). Downstream signaling from hypothalamic AgRP/NPY/GABA-secreting neurons is considered redundant, meaning that selective stimulation of individual downstream projection areas is sufficient to suppress appetite. Furthermore, cortical stimulation through these projections enhances the reward value or incentive salience of certain foods (116). Based on this neuronal projection distribution, downstream brainstem nuclei appear to regulate portion size, given their reception of vagal and circulating hormonal afferents, and can modulate food intake, digestion, and absorption (117). In contrast, the corticolimbic system (including the hippocampus, basal ganglia, and amygdala) governs the cognitive and emotional aspects of feeding behavior (118). One of the multiple theories explaining obesity focuses on the transition from high-fiber diets to energy-dense, palatable diets, with current knowledge derived mainly from animal studies and neuroimaging research (113, 119). In this context, murine studies have shown that sweet taste receptors (TAS1R2 and TAS1R3) are predominantly expressed in the hypothalamus and brainstem, whereas bitter receptors (TAS2R116, TAS2R118, TAS2R138, TAS2R104) are mainly expressed in the arcuate nucleus, ventromedial hypothalamus, dorsomedial hypothalamus, and nucleus of the solitary tract (120, 121). These animal models have also explored whether G protein-coupled taste receptor expression is altered in obesity models, particularly in leptin-deficient *ob/ob* mice and diet-induced obesity models (122). Both genetic and environmental models demonstrate reduced taste receptor expression, especially in the hypothalamus and brainstem, but no significant changes in cortical or hippocampal expression (123). In humans, various neuroimaging studies using positron emission tomography (PET) and functional magnetic resonance imaging (fMRI) have provided insights into brain responses to food stimuli (120, 124, 125). For instance, in individuals with melanocortin receptor 4 (MC4R) deficiency (a genetic model of obesity), heightened activity in the ventral and dorsal striatum has been observed in response to palatable foods compared to individuals without MC4R mutations. These findings suggest that disruption of the leptin-proopiomelanocortin-MC4R circuit is associated with reduced executive control over eating behavior (126). Since leptin reduces sweet taste sensitivity, diminished leptin signaling in the CNS may contribute to the reduced sweet taste sensitivity observed in individuals with obesity (127). Regarding fat taste, mediated by CD36, healthy individuals exhibit increased oxygen consumption in the secondary gustatory cortex (orbitofrontal and medial prefrontal cortex) following oral fat intake. Conversely, individuals with obesity show increased oxygen consumption in the insula, operculum, bilateral precuneus, and posterior cingulate cortex, along with the ventral putamen and Rolandic operculum, but decreased oxygen uptake in the lower caudate—patterns associated with increased impulsivity towards food stimuli (128–130). Moreover, amygdala responses are attenuated following the consumption of high-fat foods, reducing subsequent responses to further food stimuli (131). The magnitude of brain response to high-fat versus neutral foods has been proposed as a predictor of body mass index (BMI) in healthy volunteers (132). Concerning sweet and salty tastes, clinical studies have yielded conflicting results. Most do not find differences in sweet taste perception between individuals with and without obesity. Some studies, however, have reported decreased salty taste perception in individuals with higher BMI (133, 134). It has repeatedly been reported that an increase in BMI is associated with a reduction in the perceived intensity of taste qualities and a diminished sense of taste. Most studies point toward a reduction in taste sensitivity in obesity, which appears to be reversible with weight loss (135, 136). Taste perception influences food selection and consumption; therefore, individuals with obesity often exhibit eating behaviors characterized by the consumption of larger portions, energy-dense foods, and increased snacking behavior (104).

## 5 The role of glucagon like peptide-1 (GLP-1) in taste receptor cells

### 5.1 Overview of GLP-1 and GLP-1RAs

As it is broadly known, GLP-1 is a peptide hormone generated through the enzymatic breakdown of preproglucagon (PPG), ultimately giving rise to GLP-1 (1–37) and GLP-1 (7–36) amide or GLP-1 (7–37) peptide variants. It is synthesized in L-cells located in the intestinal mucosa, α-cells found in the pancreatic islet, and brain preproglucagon neurons residing in the NTS (137). As it is part of the G protein-coupled receptor family, it has a wide distribution through the body, predominantly localizing to the cellular membrane (138). Both GLP-1 and its agonists (GLP-1RAs) have been identified for suppressing appetite, slowing gastric emptying time, and inducing fullness and satiety, thereby reducing caloric intake under ad libitum feeding conditions (139–141). Its presence in the cardiovascular system has implications in cardiac and vascular function (142), while its expression in the central nervous system might regulate behavior, mood, and cognition (143, 144). Mostly, GLP-1RAs are primarily used as antihyperglycemic drugs. Initially approved for type 2 diabetes (T2D) treatment, they were later found to influence appetite regulation (145, 146) by regulating activity in hedonic brain regions like the insula and orbitofrontal cortex changing food preference (147), therefore, leading to their approval for obesity and diabetes management (148–150). Although all of these are considered beneficial and protective mechanisms, there is still some debate about the frequent adverse effects (AE) they have, most of them of gastrointestinal nature (151, 152). The prevalence of AE under the use of GLP-1RAs is approximately 50%, depending on the type and dose of medication; usually mentioned as mild to moderate and temporary as dose escalation is intended, so in most cases, they are resolved after the maintenance dose is reached (153). However, some people may report AE, regardless of the dose usage. Most users may experience nausea or vomiting derived by the GLP-1 activity in area postrema and delayed gastric emptying generated by the molecule (154–157). There are also motility alterations, such as diarrhea explained by the rapid colonic transit, as GLP1 activates intrinsic afferent neurons containing calcitonin gene-related peptide that favors the frequency of peristaltic contractions (158) or constipation due to mechanisms dependent on nitric oxide and AMPc/PKA that reduces colon contractility (159). Less frequent AEs like hypoglycemia, is mostly seen in combination with insulin or sulfonylureas (157) and some reports of pancreatobiliary complications are partially explained by direct altered absorption of bile acids, rapid weight loss or changes in the gallbladder motility with increased risk of pancreatitis, even though it is extremely rare (160). Consequently, dose titration is slow and dependent on AEs, and common recommendations like reduction on food quantity or high fat or spicy food is always suggested (151, 154, 158) Also, some hypotheses might suggest that GLP-1 stimulation can affect taste perception, as it has been reported by the FDA Adverse Event Reporting System (FAERS). Other symptoms, such as dysgeusia or aversion to sweet solutions and high-calorie foods can also happen in approximately 11.5% of people under GLP-1RAs treatment (161). Mechanistic studies indicate that GLP-1RAs suppress the perception of all basic taste modalities, probably through combined actions in the brainstem and vagal modulation, although paradoxical cases of sweet hypersensitivity have been documented in specific populations (162).

### 5.2 GLP-1 and its relationship with taste

**Figure 3:**
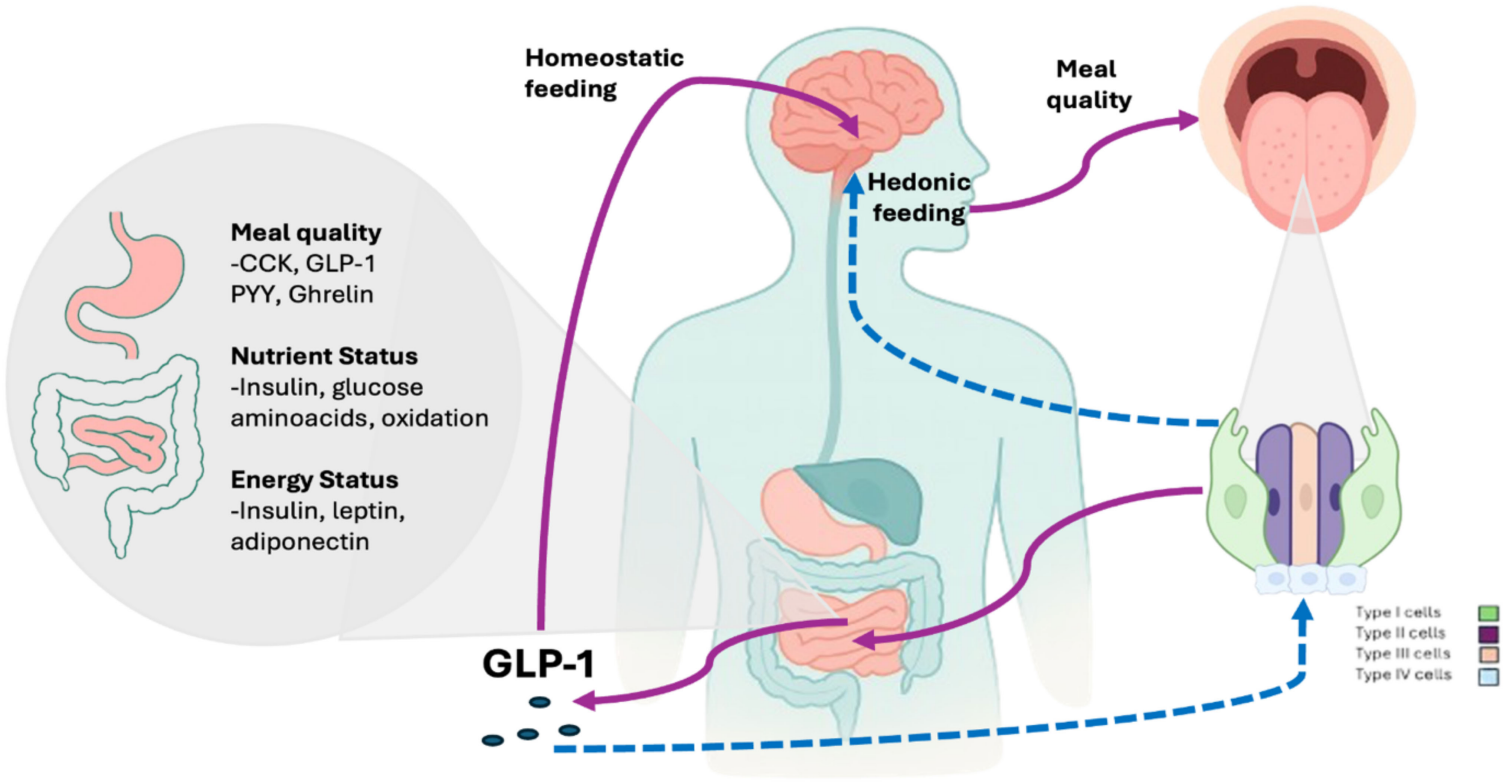
Glucagon-Like Peptide 1 (GLP1) is a peptide hormone secreted on the “L” cells in jejunum and ileum and from the cleavage of preproglucagon (PPG) in CNS in response, mainly, of glucose oral loads. Use of GLP1-RAs has shown diminished hunger by acting directly in POMC/CART (homeostatic hunger) and across the ventral tegmental area in the reward system (hedonic hunger). It is also involved across the TRC in Type II taste cells, where they modulate sweet sensitivity through the Gα-gustducin/TRPM5 pathway, and downregulate T1R2/T1R3 expression and ATP release. Also, in type III cells it may function as a neuromodulator for sweet flavor (137, 163–165, 170–172).

In recent years, the relationship between taste and GLP-1 agonism has been attempted to be elucidated, however, the exact interaction between them has not been clearly defined. The principal mechanism described is that GLP-1 signaling exerts control over gustatory perception via peripheral and central mechanisms (163). Emerging evidence demonstrates that GLP-1 receptors are expressed in Type II taste cells, where they modulate sweet sensitivity through the Gα-gustducin/TRPM5 pathway, and downregulate T1R2/T1R3 expression and ATP release (1, 15). Centrally, GLP-1 receptor activation in the NTS and ventral tegmental area (VTA) suppresses dopaminergic responses to sweet stimuli, while enhancing bitter aversion, mediated by TAS2R (164, 165). Clinically, GLP-1RAs like semaglutide induce sweet hyposensitivity and reduce preference for hyperpalatable foods, an effect mediated by changes in endogenous GLP-1 secretion and synaptic plasticity in reward circuits (166). In taste buds, GLP-1 is locally produced from proglucagon through the cleavage by prohormone convertase PC1/3, which is co-expressed with GLP-1 in all taste cells positive for this peptide. Locally, there is no DPP-4 expression in taste tissue, which could suggest that in this location, GLP-1 may achieve a longer half-life (167). Evidence suggests that GLP-1 may not act solely in a paracrine manner; recent studies show that locally secreted GLP-1 can activate GLP-1 receptor-expressing afferent fibers of the glossopharyngeal and vagal nerves, thereby transmitting peripheral gustatory information to the NTS. This establishes a direct neuroendocrine bridge between oral GLP-1 signaling and the central components of the GBA, where it converges with intestinal GLP-1-mediated pathways to regulate appetite and reward processing (58, 61, 168, 169). These findings position the entero-gustatory-endocrine axis as a therapeutic target in metabolic disorders, where GLP-1 dysfunction disrupts taste signaling, perpetuating pathological feeding behaviors (**Figure 3**).

On the other hand, immunohistochemical studies in mice may show a strong GLP-1 expression in specific subsets of taste bud cells, mainly in circumvallate and fungiform papillae, and approximately 10% of all taste cells are GLP-1 positive, possibly meaning a strong relationship with taste perception as they co-express α-gustducin, a G protein associated with the transduction of sweet, umami, and bitter tastes; conversely knockout GLP-1 -/- mice showed attenuated response to sweet flavor (61, 67, 173, 174). Also, in type III (serotonergic) cells, approximately 23% of serotonin-positive cells express GLP-1. Exposure to sugars induces rapid GLP-1 secretion from T1R3-sensitive cells, following a dose-dependent pattern where higher stimulus concentrations lead to greater secretion—an effect blocked by GLP-1 receptor antagonists (61, 67). It may be stated that it acts as a neurotransmitter or neuromodulator on adjacent GLP-1R-expressing nerve fibers, enhancing attraction to sweet tastes and contributing to residual sweet perception even in the absence of T1R3 (15, 18). GLP-1R knockout mice exhibit reduced responses to sweeteners, confirming their key role in sweet perception, as well as increased sensitivity to monosodium glutamate (MSG), suggesting GLP-1 may inhibit umami taste as well (65).

### 5.3 Clinical evidence regarding GLP-1 and taste

In the past few years, there has been growing evidence that highlights the physiological and molecular mechanisms in which GLP-1 regulates taste perception. A controlled clinical trial revealed that patients with GLP-1ARs therapy showed significant reductions in perception of all five basic tastes, scoring 29% lower on standardized tests like the Waterless Empirical Taste Test (WETT^®^) compared to controls. This study proposes that this phenomenon may arise from peripheral and central GLP-1 receptor hyperstimulation, leading to downregulation of chemosensory pathways (165). Clinical trials with liraglutide showed not only a significant increase in satiety sensation, but also a notable reduction in the desire for sugar-, fat-, and salt-rich foods, accompanied by modifications in the hormonal profile, particularly in peptide YY (PYY) levels. These changes are enhanced by modifications in key regions such as the insular cortex (main area for taste processing) and caudate nucleus, where GLP-1-RAs are capable of normalizing altered brain activation patterns in patients with obesity and diabetes mellitus when exposed to hedonic gustatory stimuli (173, 174). As well, in real-world scenarios, such as described by Brindisi et al, it is mentioned a reduction in pleasure and taste perception in overweight and people with obesity with poor type 2 diabetes control, probably by lowering the detection threshold of sweet flavors, becoming more sensitive to them, resulting in aversion for this flavor (175). In contrast, infusions of native GLP-1 at physiological doses (0.75 pmol·kg^−1^·min^−1^) show a preferential effect on the visual perception of food, without significant alterations in palatability parameters (176). More recent advances in drugs, especially semaglutide, have also been related to taste modification. Gibbons et al., studied the effect of oral semaglutide in 15 subjects with type 2 diabetes and reduction on appetite and craving, demonstrating not just the reduction in calorie intake, but also lowering the palatability and preference for sweet and high fat food (177). Also, during STEP 5, a study to assess efficacy of semaglutide with a follow-up of 2 years, there is a *post-hoc* analysis that demonstrates a better performance in the self-reported Control of Eating Questionnaire(CoEQ) to measure changes in appetite versus placebo, with lower cravings for dairy and starchy foods and less desire for salty and spicy food at short-term (20 weeks) but also as a maintenance therapy at weeks 52 and 104 (178); this finding is also aligned with the one reported from Friedrichsen et al. in which is also mentioned the improvement on this questionnaire at 20 weeks; both favoring weight loss over placebo and demonstrating the major regulation of GLP1-RA specially in people with less control over craving and hedonic hunger. It is also important to state that AEs were similar all across both studies (179). Furthermore, a recent clinical study in women with obesity treated with semaglutide showed improved recognition of basic tastes, altered gene expression in the tongue (measured by tongue biopsy), decreased activation of putamen in response to calorie-dense sweet and savory food cues, and increased cortical brain activity in response to the sweet taste stimulus compared to placebo. The potential effect of semaglutide on taste perception and recognition needs further exploration in more diverse clinical groups (180). With the introduction of dual GLP-1/GIP-RA, ongoing studies could provide novel insights and new evidence on this topic. In a phase 1 trial by Martin and colleagues, observations at week 6 comparing tirzepatide vs once-daily liraglutide and placebo have shown that the use of the latter, provides a reduction on the desire for high-fat and high-sugar food by lowering activation in the cingulate gyri, hippocampus and orbitofrontal cortex assessed by MRI and blood-oxygen-level at these areas, meaning less appetite, early satiety, and less food craving with more reduction on weight loss at short term (181). Recently, a real-world study showing association between incretin-based therapy and changes in taste perception with GLP-1 or dual GLP-1/GIP-RAs usage for around 40 weeks reported that independently to weight loss, there was an increase in sweet perception intensity up to 4.7 times and salt perception up to 3.2 times in around 20% of the participants, resulting as well in earlier satiety, and reduction of hunger and craving due to a major sensibilization to both flavors with any of these medication, although they did not mention further impact in weight loss or BMI change (170). Even the emergent evidence on this topic, there is no clear mechanism that elucidates the specific relationship between GLP-1 and GLP1-RA with taste perception, being apparently a combination of both peripheral and central actions, which are involved. Over CNS, the direct action on NTS, hypothalamic arcuate and surrounding structures, as well as the effect on the ventral tegmental area and nucleus accumbens may act on homeostatic and hedonic hunger, changing palatability and reward behavior; while the reinforcement and hypersensitivity of certain flavors and modulation of appetite by T1R and T2R families may play a peripheral action (170–172).

We summarize the clinical and preclinical evidence that includes evaluations on taste perception under GLP-1RAs treatment in **Tables 2** and **3**.

**Table 2:** Clinical evidence — GLP-1 receptor agonists, reported adverse effects and taste perception.

Author (year)	Study design	Population	Primary outcomes	Secondary outcomes	Main results	Described taste alterations
Ten Kulve et al., 2016 (174)	RCT crossover trial (GLP-1 vs Exendin 9–39 antagonist)	20 healthy adults and 20 patients with obesity with type 2 diabetes	Effect of endogenous GLP-1 and Liraglutide in CNS modulation to palatable food using fMRI	Correlations between CNS activation (insula, caudate nucleus) and weight loss	Liraglutide increased brain activation to chocolate milk in the right insula and caudate nucleus.	No direct alterations reported.
Kadouh et al., 2020 (173)	RCT (liraglutide 3 mg vs placebo, 16 weeks)	35 adults with obesity	Appetite and food preference	Body composition and gut hormones	Reduction in maximal tolerated volume with nutrient drink	Decreased liking for sweet, fatty, and salty foods
Garvey et al., 2022 (178)	***Post-hoc* analysis (STEP 5)**	304 adults with obesity	Weight loss percentage at weeks 20, 52, and 104.	Reduction on craving control and craving savory at weeks 20, 52, and 104 compared to placebo.	Weight loss -14.8% vs -2.4% with placebo	Decreased in all kinds of craving during the 104 weeks. Better weight loss at short term (20 weeks). Hypersensitivity in sweet and salt flavors.
Aldawsari et al., 2023 (176)	Systematic review (12 RCTs)	445 adults with obesity	Appetite and food preference	Weight loss and satiety	GLP-1-RAs are effective in obesity treatment	Decreased preference for sweet and fatty foods, probably not related to half-life of the agent.
Bettadapura et al., 2024 (165)	Narrative review	Human studies with GLP-1-RAs	Food choice and reward	Neural mechanisms of appetite	NA	Lowered desire for high-sugar/fat foods in humans and mice
Chen et al., 2025 (161)	Pharmacovigilance (FAERS, 28,953 reports)	GLP-1-RA users	Neurological adverse events	Onset and severity	Dizziness and Tremor were the most reported neurological adverse effects	Dysgeusia and taste disorders were more frequent in GLP-1-RAs users (ROR >1)
Martin et al., 2025 (181)	Phase 1 RCT (tirzepatide, liragutide, placebo (1:1:1 ratio))	114 adults with obesity	Appetite and intake changes vs placebo	Self-reported ingestive behavior and Brain fMRI reward response	Tirzepatide reduced energy intake (ETD -524.6 kcal) vs placebo, brain activation to high fat, high sugar food photos was decreased	Tirzepatide reduced craving for sweet and fatty foods
Mozaffarian et al., 2025 (157)	Expert consensus review	Adults on GLP-1 therapy	Nutritional and behavioral integration	Adherence and appetite	GLP-1-RA reduce body weight (5-18%) but with challenges on side effects ang high costs	Anecdotical evidence of reduced pleasure from energy-dense foods
Jensterle et al., 2025 (180)	RCT (Semaglutide 1.0 mg vs placebo, 16 weeks)	30 women with obesity and PCOS	Transcriptomic changes in tongue tissue and changes in taste sensitivity	Neural response to sweet stimuli (fMRI)	Semaglutide improved overall taste recognition (score 11.9 ± 1.9 to 14.4 ± 1.0) The genes EYA, PRMT8, CRLF1, and CYP1B1 showed differential RNA expression. Changes in putamen and angular gyrus in response to sweet solution after meal intake (semaglutide vs placebo, P <.001).	Semaglutide improved overall taste recognition.

RCT, randomized controlled trial; GLP-1, glucagon-like peptide-1; GLP-1RA, glucagon-like peptide-1 receptor agonist; CNS, central nervous system; fMRI, functional magnetic resonance imaging; PCOS, polycystic ovary syndrome; FAERS, FDA Adverse Event Reporting System; ROR, reporting odds ratio; ETD, estimated total daily energy intake; NA, not available. STEP: Semaglutide Treatment Effect in People with obesity; Post-hoc: analyses evaluated secondary behavioral outcomes beyond the prespecified primary and key secondary endpoints.

**Table 3:** Preclinical and mechanistic evidence — GLP-1 and gustatory function.

Author (year)	Model	Study design	Key findings on taste function	Mechanistic insights
Takai et al., 2015 (67)	Mouse (GLP-1R knockout)	Experimental	Loss of sweet taste response	GLP-1 enhances sweet taste via local signaling on TBC in response to hypersensitivity of T1R2/T1R3 receptors and ATP modulation by Type II cells.
Drucker et al., 2021 (147)	Human and animal	Mechanistic review	GLP-1 affects appetite and reward	Central GLP-1 receptor expressing-neurons may modulate gustatory reward both in POMC and VTA, down regulating appetite and reward (palatability).
Trapp & Brierley, 2022 (163)	Human and rodent	Neurophysiological review	GLP-1 neurons influence gustatory and reward circuits	GLP-1 by the breakdown of preproglucagon on NTS reduces dopaminergic pathways, diminishing preference for high sugar and high fat food, which implies taste and preferences are also cognitive behaviors.
Zheng et al., 2024 (139)	Translational (human/animal)	Mechanistic review	Potential modulation of taste processing and gustatory function	Integration between gut-brain and sensory pathways with GLP-1 as central coordinator.

## 6 Discussion

Recent evidence firmly establishes taste perception as a critical link between sensory biology and metabolic regulation, mediated by a complex neuroendocrine crosstalk within the gut-brain-taste axis. Far from an isolated system, the gustatory system functions as a dynamic metabolic sensor that both informs and is continuously modulated by the body’s energetic status (15, 17). Key hormones like GLP-1, leptin, ghrelin, and CCK act as crucial messengers, directly regulating the sensitivity and expression of peripheral taste receptors, while central neuromodulators, such as dopamine and serotonin, integrate these signals with reward and homeostatic circuits (172, 182).

The clinical implications of this framework are profound. Disruptions within this axis such as leptin resistance in obesity or impaired GLP-1 signaling in diabetes are not merely consequences of these diseases but active contributors to their pathogenesis by distorting taste preferences and perpetuating pathological eating behaviors. The therapeutic success of GLP-1 receptor agonists, which modulate both sweet taste perception and the hedonic valuation of food, underscores the potential of targeting this integrative system. The mechanisms underlying these effects are likely multifactorial. In the nervous system, the presence of GLP-1 receptors in the brainstem and their vagal connection suggests that the effects on taste may be secondary to central modulation rather than direct peripheral action (157, 170) but also, *in vitro* and clinical studies may suggest changes in perception of sweet, bitter and salt flavors as a reflection of GLP-1 on TBC (67, 178). Furthermore, potential crosstalk with other metabolic hormones could be a significant factor. For instance, leptin, an anorexigenic hormone, is known to suppress sweet taste sensitivity. A compelling area for future research is to investigate whether GLP-1-RAs modulate taste perception in part by altering the expression or signaling of leptin or cholecystokinin (CCK) in taste bud cells, creating an integrated hormonal milieu that fine-tunes taste sensitivity based on energy status.

Recent clinical evidence reinforces this integrative framework by showing that GLP-1RA and dual incretin agonists modify taste-related behavior through coordinated gastrointestinal, hormonal, and central mechanisms. Functional MRI trials consistently demonstrate reduced activation in mesolimbic and cortical reward circuits following treatment, reflecting diminished hedonic drive and a lower motivational response to high-calorie food cues (180, 181). Randomized trials in adults with obesity and women with PCOS further reveal decreases in liking and increased aversion toward sweet and fatty foods, supporting a shift in the hedonic valuation of food rather than a primary alteration in peripheral taste sensitivity (175–180). Additionally, real-world pharmacovigilance data highlight sensory adverse events (particularly dysgeusia) that appear more frequently in clinical practice than in controlled trials, suggesting under recognition of taste-related disturbances in research settings (159). These findings collectively indicate that the taste-modulating effects of incretin therapies arise from integrated gut-brain mechanisms that reshape reward processing and conditioned preferences.

Despite these insights, several methodological limitations constraint definitive conclusions about the direct effects of incretin therapies on taste. Most human studies rely on small sample sizes, short intervention periods, and subjective appetite or liking scales, while lacking objective gustatory measures such as taste-threshold testing or electrogustometry (153, 173, 180, 181). Such limitations make it difficult to distinguish true sensory modulation from conditioned aversion secondary to nausea or early satiety effects that are well documented across GLP-1RA trials (153, 157, 173, 180). Population diversity is also limited, with few studies including older adults, men with obesity, or individuals from different ethnic backgrounds. Moreover, null or inconsistent findings in some mechanistic trials, including absent changes in certain satiety hormones or mixed reward-response patterns, underscore the complexity of these pathways. Given that sensory or gastrointestinal adverse effects, such as dysgeusia and persistent nausea, can impair nutritional intake and lead to early treatment discontinuation (particularly in vulnerable populations) future research must incorporate rigorous sensory testing, larger and more diverse cohorts, and adherence-related outcomes to clarify how incretin therapies influence taste perception and long-term eating behavior (153, 157, 161).

The new findings in recent years raise more questions about the actual function of GLP-1 and GLP1-RA on taste regulation, like the molecular mechanisms in which the decrease or hypersensitivity of some flavors results from receptor internalization, downregulation of ion channels (such as TRPM5), alterations in neurotransmitter release (ATP/5-HT) (24, 162, 173) or either way for genetic variations and polymorphisms or if it is dependent on the density of specific subpopulations around TBC, since not all patients experience the same effect with medication (151, 158, 176). To bridge this gap, real-world data from sources like pharmacovigilance databases are invaluable. For example, a recent analysis of the FDA’s reporting system (FAERS) highlighted that taste disturbances (dysgeusia) are noted more frequently by patients using GLP-1RAs in everyday practice than in clinical trial reports (161). Moving forward, a concerted effort to include more representative populations in research is essential to fully understand how GLP-1-based treatments influence taste perception across all demographics. Furthermore, a critical appraisal of the cited evidence reveals recurrent limitations. Many human RCTs included in this review (e.g., Kadouh et al., 2020; Jensterle et al., 2025) feature small sample sizes, which limit statistical power and generalizability. Intervention periods are often short-term, obscuring the long-term dynamics of taste modulation. Translation from preclinical models is complicated by fundamental differences in taste physiology and GLP-1 signaling pathways between rodents and humans. Importantly, the narrative is balanced by acknowledging null findings, such as the study by Shirazi-Beechey et al. (68), which reported that sweet taste receptor antagonism with lactisole reduced GLP-1 and PYY secretion but had no effect on CCK release, highlighting pathway-specific effects.

Gustatory sense remains one of the most intriguing sensory systems. Despite long-standing study, its characterization from an endocrinological perspective is incomplete. Taste receptor cells play a dual role, and we now know their participation extends far beyond the tongue and oral cavity. It is possible that subpopulations of these cells exist, each with distinct roles in energy homeostasis. The use of GLP-1-RAs and other incretin-based therapies has proven effective not only by reducing food intake but also by fundamentally altering food preferences. While mechanisms have been proposed, the full picture, including potential hormonal crosstalk and systemic influences like the gut-microbiota-brain axis, warrants dedicated future research. As we navigate an obesogenic environment and confront obesity as a complex disease, the continued development of therapeutics that target gut peptides and taste receptors will undoubtedly play a major role in the future of metabolic medicine.

## 7 Conclusion and future directions

Future research should focus on translating preclinical findings to human applications, particularly through chronobiological studies of hormonal-taste interactions and advanced computational modeling of individual taste-metabolism relationships. The convergence of sensory science with precision medicine holds particular promise for developing more effective, targeted interventions for metabolic diseases, where optimizing taste perception could significantly improve dietary adherence and treatment outcomes while minimizing side effects. Deeper understanding of these mechanisms may revolutionize the therapeutics of obesity, diabetes, and related disorders through interventions that harmonize sensory experience with metabolic needs. Despite recent evidence, there is still a gap on this topic, in which there is a clear need for studies on long term, bigger samples and studies not only based on subjective scales, but actually, including the complex interplay of gut hormones, molecular profile and combination with neuroimaging for a better understanding of the GBA. The inclusion in another largely unexplored dimension, as the potential role it may have on the gut-microbiota-brain axis is also interesting. Given that GLP-1-RAs alter gut motility and secretion, and that the gut microbiota can influence host eating behavior, including food preferences, it is plausible that these drugs indirectly influence taste perception by modulating microbial composition and the subsequent production of metabolites that can signal to the brain, potentially via vagal afferents or systemic circulation. Looking forward, the field is moving toward precision medicine. Individual genetic profiles of taste receptors, circadian rhythms in hormonal sensitivity, and personalized microbiome-gustatory interactions will pave the way for tailored interventions. These could range from pharmacotherapies targeting specific taste receptors (e.g., TAS2R agonists) to chrono-nutritional strategies and neuromodulation techniques designed to restore healthy signaling in pathological states. Finally, harmonizing the sensory experience with metabolic needs by modulating this axis emerges as a promising and transformative strategy for managing metabolic disorders in the 21st century.
